# A Mathematical Model to Predict Diagnostic Periods for Secondary Distant Metastases in Patients with ER/PR/HER2/Ki-67 Subtypes of Breast Cancer

**DOI:** 10.3390/cancers12092344

**Published:** 2020-08-19

**Authors:** Ella Ya. Tyuryumina, Alexey A. Neznanov, Jacob L. Turumin

**Affiliations:** 1 International Laboratory for Intelligent Systems and Structural Analysis, Faculty of Computer Science, National Research University Higher School of Economics, 109028 Moscow, Russia; aneznanov@hse.ru; 2 Medical Center “For all Family”, 664047 Irkutsk, Russia; yatyuryumin@gmail.com

**Keywords:** breast cancer, secondary distant metastases, survival, tumor volume doubling time, ER/PR/HER2/Ki-67, mathematical model

## Abstract

Previously, a consolidated mathematical model of primary tumor (PT) growth and secondary distant metastasis (sdMTS) growth in breast cancer (BC) (CoMPaS) was presented. The aim was to detect the diagnostic periods for visible sdMTS via CoMPaS in patients with different subtypes ER/PR/HER2/Ki-67 (Estrogen Receptor/Progesterone Receptor/Human Epidermal growth factor Receptor 2/Ki-67 marker) of breast cancer. CoMPaS is based on an exponential growth model and complementing formulas, and the model corresponds to the tumor-node-metastasis (TNM) staging system and BC subtypes (ER/PR/HER2/Ki-67). The CoMPaS model reflects (1) the subtypes of BC, such as ER/PR/HER2/Ki-67, and (2) the growth processes of the PT and sdMTSs in BC patients without or with lymph node metastases (MTSs) in accordance with the eighth edition American Joint Committee on Cancer prognostic staging system for breast cancer. CoMPaS correctly describes the growth of the PT in the ER/PR/HER2/Ki-67 subtypes of BC patients and helps to calculate the different diagnostic periods, depending on the tumor volume doubling time of sdMTS, when sdMTSs might appear. CoMPaS and the corresponding software tool can help (1) to start the early treatment of small sdMTSs in BC patients with different tumor subtypes (ER/PR/HER2/Ki-67), and (2) to consider the patient almost healthy if sdMTSs do not appear during the different diagnostic periods.

## 1. Introduction

After the primary tumor (PT) of breast cancer (BC) is diagnosed, multimodal treatment occurs, including surgery (lumpectomy, unilateral or bilateral mastectomy), radiotherapy, and chemotherapy [1]. However, different parameters, such as the size of the PT, the number of affected lymph nodes, and the growth rate of metastases (MTSs), influence the appearance of secondary distant metastases (sdMTSs) in different organs [2,3,4,5,6,7,8,9,10,11,12,13,14,15]. Moreover, these different parameters determine the period from resection of the PT to the first clinical manifestation of sdMTSs (MTS-free survival time or non-visible period) [8,9,13,16,17,18,19,20,21]. Although the interval of time from the date of diagnosis (using the tumor-node-metastasis (TNM) staging system) to the date of the patient’s death is referred to as survival (lifetime), it is commonly defined as a rate per hundred living patients for a certain period after the diagnosis [1,10,11,14,15]. Hence, survival includes the non-visible growth period (MTS-free period) and the visible growth period of sdMTSs, diagnostics, treatment, and patient death [21]. PTs of breast cancer grow at different rates: rapid, intermediate, and slow [2,3,4,6,7]. The growth rate of sdMTSs depends on the growth rate of the PT [2,3,4,6,7]. The growth rate of PTs in BC may depend on different subtypes of ER/PR/HER2/Ki-67 (Estrogen Receptor/Progesterone Receptor/Human epidermal growth factor Receptor 2/Ki-67 marker) expression in BC [22,23,24,25,26].

The current guidelines on BC follow-up recommend regular visits. The European Society of Medical Oncology (ESMO) recommends follow-up visits every 3–4 months after resection of the PT for the first two years [27]. The American Cancer Society/American Society of Clinical Oncology (ASCO) recommends follow-up visits every 3–6 months after resection of the PT for the first three years, every 6–12 months for the next two years, and every 12 months after the first five years [28].

The National Comprehensive Cancer Network (NCCN) recommends follow-up visits every 4–6 months after resection of the PT for the first five years and every 12 months after the first five years [29]. The Associazione Italiana di Oncologia Medica (AIOM) recommends follow-up visits every 3–6 months after resection of the PT for the first five years and every 12 months after the first five years [29].

The guidelines recommend performing annual examinations, including bilateral (in the case of organ-preserving surgery) or contralateral mammography, a computed tomography of the thoracic organs, and an ultrasound examination of the abdominal organs [1,27,28,29,30,31,32].

All BC patients receive comprehensive PT treatment. The duration of the sdMTS-free period depends equally on both the metastatic tumor rate and the duration of the preclinical non-visible growth period of BC (doubling time) [7,21,33,34,35,36,37,38,39,40,41,42].

Case studies from the BC field show that mathematical models can provide tangible advantages [7,10,11,12,14,15,16,17,18,19,20,21,33,34,35,36,37,38,39,40,41,42]. For instance, a consolidated mathematical model of PT growth and sdMTS growth in BC (CoMPaS) allows the calculation of the number of doublings and the tumor volume doubling time (TVDT) (days) for the different growth periods throughout the whole BC process [21].

The current guidelines for multimodal examinations are generalized to all patients [43]. Consequently, there is a lack of personalized recommendations for multimodal examinations to detect sdMTSs in patients with BC with regard to the stage and/or the growth rate of the PT. All patients with BC worry about the appearance of sdMTSs after resection of the PT. Thus, the most important question is whether sdMTSs will appear.

If sdMTSs do appear, when will the sdMTSs first materialize? The available clinical studies provide no information about the period of manifestation of sdMTSs after PT resection in each patient as a function of the size of the PT and the stage of the BC. The problem becomes very complex because the patients must obtain a personalized approach to build a schedule of multimodal examinations to detect sdMTSs at the early stage and to start early treatment, which can increase the patient’s lifetime. Currently, the possibility of calculating the earliest diagnostic period of sdMTSs in patients with BC, taking into account the stage and/or the growth rate of the PT, was not proposed in any mathematical model in the available studies.

Moreover, if sdMTSs do not appear, when will the patient be considered healthy? Currently, there is no answer to this question.

The purpose of this study was to answer these questions using the model CoMPaS, considering the TNM stage and ER/PR/HER2/Ki-67 subtypes. Therefore, the aim of the research was to detect the personalized diagnostic periods for visible sdMTS via CoMPaS in patients with different subtypes (ER/PR/HER2/Ki-67) of breast cancer. The personalized diagnostic periods for multimodal examinations during the MTS-free period were calculated in BC patients depending on the TVDT of PT and TVDT of sdMTSs.

## 2. Results

### 2.1. Consolidated Mathematical Growth Model of Primary Tumor and Secondary Distant Metastases (CoMPaS) in Patients with ER/PR/HER2/Ki-67 Subtypes and Stage I/II/III of Breast Cancer with or without Metastases in the Lymph Nodes

**Figure 1:**
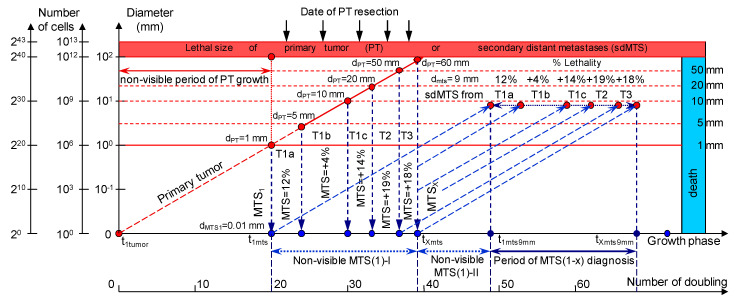
The mathematical model used to predict the earliest diagnostic period of the secondary distant metastasis (sdMTS) in patients with breast cancer (BC). T1a = 1 mm < d ≤ 5 mm; T1b = 5 mm < d ≤ 10 mm; T1c = 10 mm < d ≤ 20 mm; T2 = 20 mm < d ≤ 50 mm; T3 = d > 50 mm [14]. *Non-visible MTS(1)-I* (years)—the non-visible growth period of the sdMTS (the first non-visible sdMTS from the primary tumor (PT)) can be calculated as the period from the appearance of the first metastatic cell of the sdMTS (d = 10 µm) to the detection of the non-visible sdMTS (before the date of PT surgery); *Non-visible MTS(1)-II* (years)—the non-visible growth period of sdMTS (the first non-visible sdMTS from the PT) can be calculated as the period from the diagnosis (after date of PT surgery) to the diagnosis of the visible size (d = 9 mm) of at least one sdMTS; *Visible MTS(1)* (years)—the visible growth period of sdMTS(1) (the first visible sdMTS from the PT) can be calculated as the period from the diagnosis of the visible size (d = 9 mm) to when it reaches the lethal size (death); *Visible MTS(X)* (years)—the visible growth period of sdMTS(X) (the last visible sdMTS from the PT) can be calculated as the period from the diagnosis of the visible size (d = 9 mm) to when it reaches the lethal size (death); *Period of MTS(1-X) diagnosis*—the period of the diagnosis of the visible size (d = 9 mm) of the sdMTS from the first visible sdMTS (1) to the last visible sdMTS (X).

The CoMPaS model helps to determine the causes of BC appearance, which can lead to the development of prevention methods and a deeper understanding of the BC process (Figure 1; Table 1) [21].

sdMTSs are formed from the metastatic cells of the metastasizing PT. The diameter of the metastasizing PT may vary in size from 1 mm up to the diameter of the PT at the time of resection (non-visible MTS-I period) (Figure 1).

#### 2.1.1. T1cN0-3M0. The Whole Natural History of the PT (Triple-Negative, HR(−)/HER2(−), Ki-67 ≥ 14) and sdMTS

**Figure 2:**
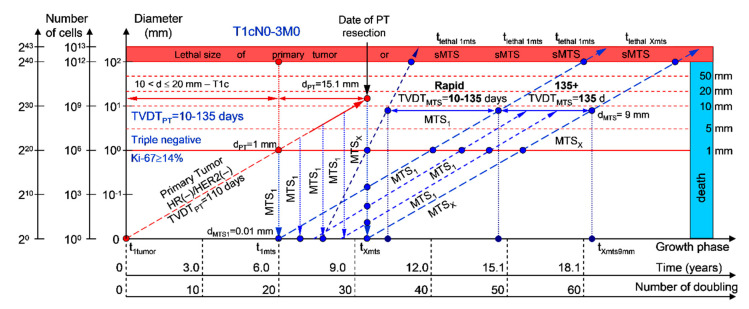
T1cN0-3M0. The *whole natural history* of the PT (triple-negative, HR(−)/HER2(−), Ki-67 ≥ 14) and sdMTS. Parameter T1c: 10 mm < d ≤ 20 mm. Diameter of PT = d_PT_ = 15.1 mm, TVDT_PT_ = 10–135 days. Rapid growth rate of secondary distant MTS = TVDT_MTS_ = 10–135 days. Mean TVDT_MTS_ = 40–67 days.

If the diameter of the PT at its resection, d_PT_, was 15.1 mm (Figure 2; Table 1), and the TVDT_PT_ was 10–135 days (a rapid growth rate of PT in patients with HR(−)/HER2(−) (triple-negative) tumors), then the diameter of the sdMTS at PT resection, d_MTS_, could be 0.01–1.00 mm.

For patients in the rapid growth rate group, TVDT_MTS_ was equal to 10–135 days. The non-visible growth period of sdMTS(1-X)-I was 0.55–4.34 years (Figure 2; Table 1). The non-visible growth period of sdMTS(1-X)-II was 0.26–6.56 years. The visible growth period of sdMTS(1-X) was 0.29–3.90 years. The survival of patients with BC was 0.55–10.47 years.

In summary, in this period of rapid growth rate of sdMTS, patients with T1cN0-3M0 BC must undergo multimodal examination every three months 0.26 years after resection of the PT for 6.30 years.

The total period of MTS(1-X) diagnosis may be 10.64 years (Figure 2; Table 1). BC patients with d_PT_ = 15.1 mm and TVDT_PT_ = 10–135 days (T1aN0-3M0) may be considered healthy only after 10.64 years if sdMTSs were not diagnosed in this period.

#### 2.1.2. T1cN0-3M0. The Whole Natural History of the PT (HER2-Positive, HR(−)/HER2(+), Ki-67 ≥ 14) and sdMTS

**Figure 3:**
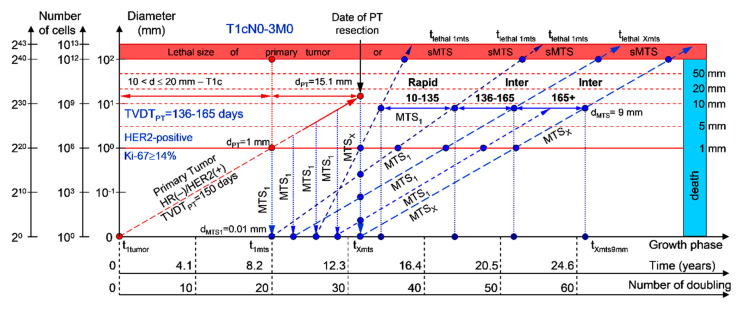
T1cN0-3M0. The *whole natural history* of the PT (HER2-positive, HR(−)/HER2(+), Ki-67 ≥ 14) and sdMTS. Parameter T1c: 10 mm < d ≤ 20 mm. Diameter of primary tumor = d_PT_ = 15.1 mm, TVDT_PT_ = 136–165 days. Rapid growth rate of secondary distant MTS = TVDT_MTS_ = 10–135 days. Intermediate growth rate of secondary distant MTS = TVDT_MTS_ = 136–165 days. Mean TVDT_MTS_ = 68–82 days.

If the diameter of the PT at its resection, d_PT_, was 15.1 mm (Figure 3; Table 1), and TVDT_PT_ was 136–165 days (intermediate growth rate of PT in patients with HR(−)/HER2(+) (HER2-positive) tumors), then the diameter of the sdMTS at PT resection, d_MTS_, could be 0.01–1.00 mm. TVDT_MTS_ was equal to 10–135 days for rapid growth rates and/or 136–165 days for intermediate growth rates.

The total period of MTS(1-X) diagnosis may be 13.07 years (Figure 3; Table 1). BC patients with d_PT_ = 15.1 mm and TVDT_PT_ = 136–165 days (T1aN0-3M0) may be considered healthy only after 13.07 years if sdMTSs were not diagnosed in this period.

#### 2.1.3. T1cN0-3M0. The Whole Natural History of the PT (Luminal B, HR(+)/HER2(−), Ki-67 ≥ 14) and sdMTS

**Figure 4:**
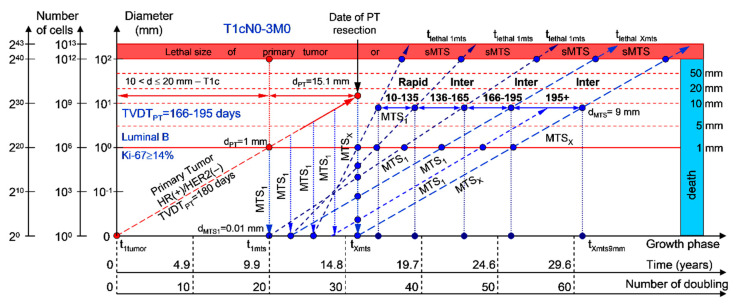
T1cN0-3M0. The *whole natural history* of the PT (Luminal B, HR(+)/HER2(−), Ki-67 ≥ 14) and sdMTS. Parameter T1c: 10 mm < d ≤ 20 mm. Diameter of primary tumor = d_PT_ = 15.1 mm, TVDT_PT_ = 166–195 days. Rapid growth rate of secondary distant MTS = TVDT_MTS_ = 10–135 days. Intermediate growth rate of secondary distant MTS = TVDT_MTS_ = 136–195 days. Mean TVDT_MTS_ = 83–97 days.

If the diameter of the PT at its resection, d_PT_, was 15.1 mm (Figure 4; Table 1), and the TVDT_PT_ was 166–195 days (intermediate growth rate of PT in patients with the luminal B subtype (HR(+)/HER2(−))), then the diameter of the sdMTS at the PT resection, d_MTS_, could be 0.01–1.00 mm. TVDT_MTS_ was equal to 10–135 days for rapid growth rates, 136–165 days for intermediate growth rates, and/or 166–195 days for intermediate growth rates.

The total period of MTS(1-X) diagnosis may be 15.49 years (Figure 4; Table 1). BC patients with d_PT_ = 15.1 mm and TVDT_PT_ = 166–195 days (T1aN0-3M0) may be considered healthy only after 15.49 years if sdMTSs were not diagnosed in this period.

#### 2.1.4. T1cN0-3M0. The Whole Natural History of the PT (Luminal B, HR(+)/Her2(+), Ki-67 ≥ 14) and sdMTS

**Figure 5:**
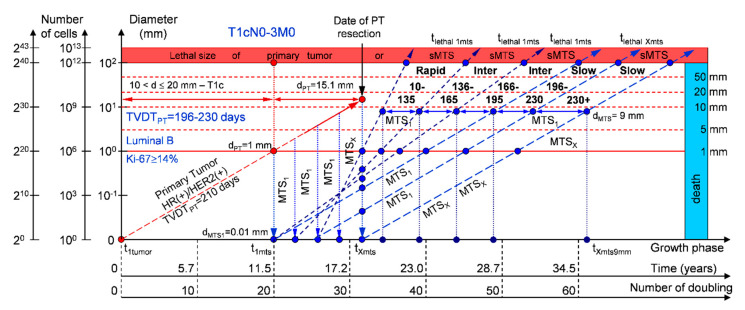
T1cN0-3M0. The *whole natural history* of the PT (Luminal B, HR(+)/Her2(+), Ki-67 ≥ 14) and sdMTS. Parameter T1c: 10 mm < d ≤ 20 mm. Diameter of primary tumor = d_PT_ = 15.1 mm, TVDT_PT_ = 196–230 days. Rapid growth rate of secondary distant MTS = TVDT_MTS_ = 10–135 days. Intermediate growth rate of secondary distant MTS = TVDT_MTS_ = 136–195 days. Slow growth rate of secondary distant MTS = TVDT_MTS_ = 196–230 days. Mean TVDT_MTS_ = 98–115 days.

If the diameter of the PT at its resection, d_PT_, was 15.1 mm (Figure 5; Table 1), and TVDT_PT_ was 196–230 days (slow growth rate of PT in patients with the luminal B subtype (HR(+)/HER2(+))), then the diameter of the sdMTS at PT resection, d_MTS_, could be 0.01–1.00 mm. TVDT_MTS_ was equal to 10–135 days for rapid growth rates, 136–165 days for intermediate growth rates, 166–195 days for intermediate growth rates, and/or 196–230 days for slow growth rates.

The total period of MTS(1-X) diagnosis may be 18.32 years (Figure 5; Table 1). BC patients with d_PT_ = 15.1 mm and TVDT_PT_ = 196–230 days (T1aN0-3M0) may be considered healthy only after 18.32 years if sdMTSs were not diagnosed in this period.

#### 2.1.5. T1cN0-3M0. The Whole Natural History of the PT (Luminal A, HR(+)/Her2(−), Ki-67 < 14) and sdMTS

**Figure 6:**
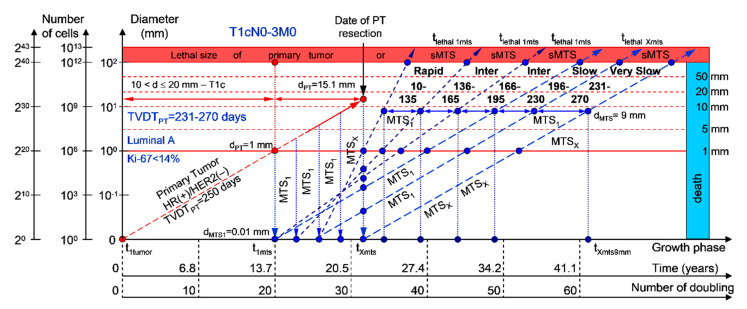
T1cN0-3M0. The *whole natural history* of the PT (Luminal A, HR(+)/Her2(−), Ki-67 < 14) and sdMTS. Parameter T1c: 10 mm < d ≤ 20 mm. Diameter of primary tumor = d_PT_ = 15.1 mm, TVDT_PT_ = 231–270 days. Rapid growth rate of secondary distant MTS = TVDT_MTS_ = 10–135 days. Intermediate growth rate of secondary distant MTS = TVDT_MTS_ = 136–166–195 days. Slow growth rate of secondary distant MTS = TVDT_MTS_ = 196–230 days. Very slow growth rate of secondary distant MTS = TVDT_MTS_ = 231–270 days. Mean TVDT_MTS_ = 116–135 days.

If the diameter of the PT at its resection, d_PT_, was 15.1 mm (Figure 6; Table 1), and TVDT_PT_ was 231–270 days (very slow growth rate of the PT in patients with the luminal A subtype (HR(+)/HER2(−))), then the diameter of the sdMTS at PT resection, d_MTS_, could be 0.01–1.00 mm. TVDT_MTS_ was equal to 10–135 days for rapid growth rates, 136–165 days for intermediate growth rates, 166–195 days for intermediate growth rates, 196–230 days for slow growth rates, and/or 231–270 days for very slow growth rates.

The total period of MTS(1-X) diagnosis may be 12.87 years (without a 270+ period) (Figure 6; Table 1). BC patients with d_PT_ = 15.1 mm and TVDT_PT_ = 231–270 days (T1aN0-3M0) may be considered healthy only after 12.87 years if sdMTSs were not diagnosed in this period.

The CoMPaS model may help calculate the non-visible MTS-II period (MTS-free period) (Figure 2, Figure 3, Figure 4, Figure 5 and Figure 6; Table 1). These calculated data may help define the minimal number of patient examinations in the non-visible MTS-II period (MTS-free period) depending on the growth rate of the PT and sdMTS (rapidly growing MTS, intermediately growing MTS, and slowly and very slowly growing MTS) (Figure 2, Figure 3, Figure 4, Figure 5 and Figure 6; Table 1).

#### 2.1.6. Examinations during the MTS-Free Period

Therefore, every patient obtains personalized data on an adequate minimal number of examinations during the MTS-free period (non-visible MTS-II period). Examples are as follows:

In the period of a rapid growth rate of sdMTSs (group V with subtype V of BC—triple-negative tumors = HR(−)/HER2(−)), patients must undergo multimodal examination every three months;

In the period of an intermediate growth rate of sdMTSs (group IV with subtype V of BC—HER2-positive tumors = HR(−)/HER2(+)), patients must undergo multimodal examination every five months;

In the period of an intermediate growth rate of sdMTSs (group III with subtype III of BC—luminal B = HR(+)/HER2(−)), patients must undergo multimodal examination every six months;

In the period of a slow growth rate of sdMTSs (group II with subtype II of BC—luminal B = HR(+)/HER2(+)), patients must undergo multimodal examination every eight months;

In the period of a very slow growth rate of sdMTSs (group I with subtype I of BC—luminal A = HR(+)/HER2(−)), patients must undergo multimodal examination every nine months.

Hence, every patient obtains personalized data on an adequate maximal quantity of examinations during this period for the early diagnosis of visible sdMTSs (diameter = 5–9 mm). This may help oncologists start early treatment of small sdMTSs in BC patients (T1-3N0-3M0) and increase the survival of BC patients with sdMTSs (T1-3N0-3M0).

The CoMPaS model calculates the different diagnostic periods of sdMTS in patients with BC (T1-3N0-3M0) and facilitates the understanding of the periods of appearance and materialization of sdMTSs.

### 2.2. Application of Consolidated Mathematical Growth Model of Primary Tumor and Secondary Distant Metastases (CoMPaS) in Patients with ER/PR/HER2/Ki-67 Subtypes and Stage I/II/III Breast Cancer in Clinical Practices

This mathematical model used to predict the different diagnostic periods of sdMTS in patients with BC may help explain difficult clinical cases of BC patient survival (Figure 2, Figure 3, Figure 4, Figure 5 and Figure 6; Table 1).

The 5–15-year survival of patients with BC depends on the diameter of the PT at resection and TVDT_MTS_ from 10 to 270 days (Figure 2, Figure 3, Figure 4, Figure 5 and Figure 6; Table 1).

**Figure 7:**
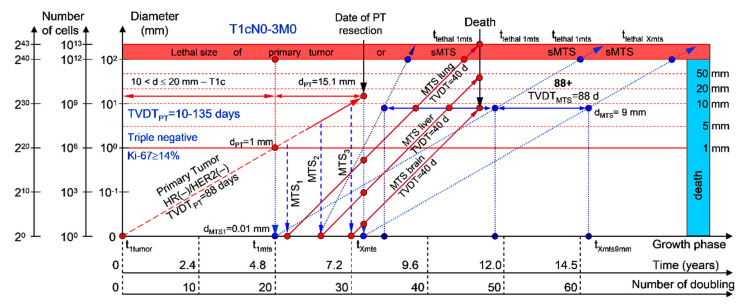
T1cN0-3M0. The *whole natural history* of the PT (triple-negative, HR(−)/HER2(−), Ki-67 ≥ 14) and sdMTSs of patient with multiple single MTSs (lung, liver, brain). Parameter T1c: 10 mm < d ≤ 20 mm. Diameter of primary tumor = d_PT_ = 15.1 mm, TVDT_PT_ = 88 days. Rapid growth rate of secondary distant MTS: TVDT_MTS_ of lung metastasis = 40 days, TVDT_MTS_ of liver metastasis = 40 days, TVDT_MTS_ of brain metastasis = 40 days.

**Figure 8:**
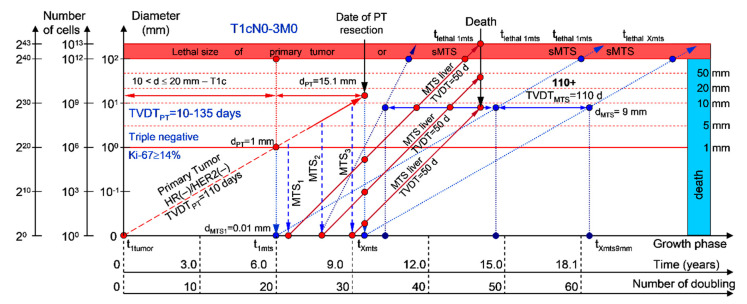
T1cN0-3M0. The *whole natural history* of the PT (triple-negative, HR(−)/HER2(−), Ki-67 ≥ 14) and sdMTSs of patient with multiple MTSs (liver). Parameter T1c: 10 mm < d ≤ 20 mm. Diameter of primary tumor = d_PT_ = 15.1 mm, TVDT_PT_ = 110 days. Rapid growth rate of secondary distant MTS: TVDT_MTS_ of liver metastasis = 50 days.

Moreover, if patients with BC have a TVDT_PT_ from 10 to 135 days (rapid growth rate of the PT—group V with subtype V of BC—triple-negative tumors = HR(−)/HER2(−)), they have a high risk of death within five years (rapid (TVDT_MTS_ = 10–135 days) growth rate of sdMTSs) (Figure 7 and Figure 8).

If patients with BC have a TVDT_PT_ from 136 to 165 days (intermediate growth rate of PT), they have an intermediate risk of death between five and 10 years (rapid (TVDT_MTS_ = 10–131 days) and/or intermediate (TVDT_MTS_ = 136–165 days) growth rate of sdMTSs).

If patients with BC have a TVDT_PT_ from 166 to 195 days (intermediate growth rate of PT), they have a low risk of death during the period from 10 to 15 years (intermediate (TVDT_MTS_ = 135–165 days or TVDT_MTS_ = 166–195 days) growth rate of sdMTSs).

If patients with BC have a TVDT_PT_ from 196 to 230 days (slow growth rate of PT), they have a low risk of death during the period from 15 to 20 years (intermediate (TVDT_MTS_ = 135–165 days or TVDT_MTS_ = 166–195 days) and/or slow (TVDT_MTS_ = 196–230 days) growth rate of sdMTSs).

If patients with BC have a TVDT_PT_ from 231 to 270 days (very slow growth rate of PT), they have a low risk of death during the period from 20 to 25 years (intermediate (TVDT_MTS_ = 135–165 days or TVDT_MTS_ = 166–195 days) or slow (TVDT_MTS_ = 196–230 days) or very slow (TVDT_MTS_ = 231–270 days) growth rate of sdMTSs).

### 2.3. Software Tool for Personalized Scheduling of Multimodal Examinations

CoMPaS was updated with a feature that includes a recommendation to have a multimodal examination every 3–4 months k_1_ years after resection of the PT for k_2_ years. The recommendation can help oncologists assign early treatment and increase survival.

## 3. Discussion

### 3.1. The Relationship between the Size of the PT and an Appearance of the sdMTSs and TVDT

Previous studies showed that (a) the mortality rate depends directly on the PT size and (b) the risk of sdMTS appearance depends directly on the PT size [2,3,4,5,6,7,8,9,10,11,12,13,14,15,16,17,18,19,20,21,36,37,38,39,40,73,74,75,76,77,78,79,80,81,82,83,84,85,86,87,88,89].

In 2010, Holzel et al. [14] and Engel et al. [10,11,15] studied the 10- and 18-year survival from the diagnosis date to PT resection using a large patient group (*n* = 33475) with regard to the BC stage (parameter T—size of the PT). It was demonstrated that the 10-year mortality linearly increases with the increasing diameter of the PT [15]. In patients with stage pT3, the 18-year mortality was higher than that in patients with stage pT2 and so forth, i.e., the 18-year mortality was much higher in patients with pT3 > pT2 > pT1c > pT1b > pT1a [14].

Hence, a larger PT results in a longer period from the determination of a visible size of the PT to the presurgery size, as well as more time for sdMTS formation before PT resection (Figure 1). In contrast, a smaller PT results in a shorter visible growth period of the PT, as well as less time for sdMTS formation before PT resection (Figure 1) [2,3,4,5,6,7,8,9,10,11,12,13,14,15,20,21,36,37,38,39,40,73,74,75,76,77,78,79,80,81,82,83,84,85,86,87,88,89].

The duration of the non-visible MTS-I period depends on the number of doublings and the TVDT [21]. According to early research results from a sequence of sdMTS appearances in BC patients after multimodal therapy, (1) a total of 75% of the recurrences were found in the first five years, (2) a total of 20% of the recurrences were found between 5–15 years, and (3) the remaining 5% of the recurrences were found in 15–25 years [20]. Therefore, the growth rate of the metastatic tumor can increase 2.2 times that of the PT; meanwhile, the TVDT can decrease 2.2 times [20].

### 3.2. The Relationship between the TVDT and the Subtypes ER/PR/HER2/Ki-67 of BC

The following observations were made: (1) BC patients with axillary lymph node MTS have a shorter TVDT than BC patients without axillary lymph node MTS (*p* < 0.05); (2) BC patients positive for the estrogen receptor ER(+) in the PT have a longer TVDT than BC patients negative for the ER(−) in the PT (*p* < 0.05); (3) BC patients positive for the progesterone receptor PR(+) in the PT have a longer TVDT than BC patients negative for the PR(−) in the PT (*p* < 0.05); (4) BC patients negative for the Ki-67(−) receptor in the PT have a longer TVDT than BC patients positive for the Ki-67(+) receptor in the PT (*p* < 0.05); (5) BC patients positive for HER2(+) in the PT have a much longer TVDT than BC patients with triple-negative HR(−)/HER2(−) expression in the PT (*p* < 0.05) [52]. In other words, this study proposes that BC patients with regional lymph node MTSs have more aggressive BC and a higher risk for the appearance of sdMTSs than patients without regional lymph node MTSs who have a lower risk for the appearance of sdMTSs.

In addition, BC patients with triple-negative (HR(−)/HER2(−)) BC have more aggressive BC and a higher risk of sdMTS appearance than BC patients positive for the progesterone receptor PR(+) who have a lower risk of sdMTS appearance. Ryu et al. [49] reported the following: (1) BC patients positive for ER in the PT (ER(+)) and BC patients positive for HER2 in the PT (HER2(+)) have a much longer TVDT than BC patients with triple-negative expression in the PT (*p* < 0.05).

The TVDT is one of the most critical parameters used to develop the consolidated mathematical growth model of the PT and sdMTSs of BC (CoMPaS) and to calculate the earliest diagnostic period of sdMTSs in patients with BC [21]. The TVDT, which helped in the development of the mathematical growth model, is a combined quality indicator that reflects the subtype of BC, the proliferative activity, the degree of tumor differentiation, the Nottingham prognostic index (NPI) score, receptor activity (ER(+), ER(−), PR(+), PR(−), HER2(+), HER2(−), Ki-67), and triple-negative BC [47,48,49,50,51,52,53,54,63,64,65,66,67,68,69,70,71,72,90,91,92,93,94,95,96,97,98,99,100]. Hence, patients can obtain a personalized approach for building a schedule of multimodal examinations to detect sdMTSs at the early stage and to start early treatment that can increase the patient’s life [43].

### 3.3. The Relationship between the Different Diagnostic Periods and Subtypes ER/PR/HER2/Ki-67 of BC

Mathematical models could lead to more precise results and more meaningful prognostic risk scores, and they could integrate the planning of multimodal examinations into outcome-oriented clinical decision-making. While many useful stand-alone models are already in clinical use for forecasting the development of the BC process, the following question remains: will the patient have sdMTSs? If yes, when will the earliest period of the clinical manifestation of sdMTSs occur? If no, when will the patient be considered healthy? To answer these questions, CoMPaS was chosen as the main research tool.

Consequently, this study concentrated on calculating the different diagnostic periods (rapid growth rates in patients with triple-negative tumors (HR(−)/HER2(−)), intermediate growth rates in patients with HER2-positive tumors (HR(−)/HER2(+)), intermediate growth rates in patients with luminal B tumors (HR(+)/HER2(−)), slow growth rates in patients with luminal B tumors (HR(+)/HER2(+)), and very slow growth rates in patients with luminal A tumors (HR(+)/HER2(−))) before the manifestation of sdMTSs (MTSs) after resection of the PT. The broad implementation of the model in everyday oncology requires versatile software platforms that can be easily integrated into existing workflows and information technology (IT) architectures. Therefore, the CoMPaS model was integrated into an iOS application with input parameters such as patient data from examinations and was updated with the possibility of calculating the earliest diagnostic period.

The consolidated mathematical growth model (CoMPaS) and the corresponding software tool may be used for work with the eighth edition American Joint Committee on Cancer (AJCC) prognostic staging system for breast cancer [69,70,71,72].

If a patient has TVDT_PT_ data, the consolidated mathematical growth model (CoMPaS) helps to calculate the data for the new personalized screening program of the sdMTS of breast cancer for each patient with T3N0-3M0 and the ER/PR/HER2/Ki-67 subtypes depending on the natural growth rate of the PT and MTS (the diagnostic periods of rapidly growing sdMTSs (HR(−)/HER2(−)—triple-negative tumors), intermediately growing sdMTSs (HR(−)/HER2(+)—HER2-positive tumors), intermediately growing sdMTSs (luminal B = HR(+)/HER2(−)), slowly growing sdMTSs (luminal B = HR(+)/HER2(+)), and very slowly growing sdMTSs (luminal A = HR(+)/HER2(−))) (Figure 1, Figure 2, Figure 3, Figure 4, Figure 5, Figure 6, Figure 7 and Figure 8; Table 1) [22,23,69,70,71,72,101,102,103,104,105,106].

### 3.4. The Relationship between the Mortality and the Convalescence and Subtypes ER/PR/HER2/Ki-67 of BC

In patients with triple-negative tumors (HR(−)/HER2(−)), the five-year mortality was higher than that in patients with HER2-positive tumors (HR(−)/HER2(+)) and so forth, i.e., the five-year mortality was much higher in patients with triple-negative tumors (HR(−)/HER2(−)) > patients HER2-positive tumors (HR(−)/HER2(+)) > patients with luminal B subtype tumors (HR(+)/HER2(−)) > patients with luminal B subtype tumors (HR(+)/HER2(+)) > patients with luminal A subtype tumors (HR(+)/HER2(−)) [22,23,24,25,26,69,70,71,72,101,102,103,104,105,106].

Moreover, if sdMTSs did not appear in the different diagnostic periods (rapid growth rate in patients with triple-negative tumors (HR(−)/HER2(−)), intermediate growth rate in patients with HER2-positive tumors (HR(−)/HER2(+)), intermediate growth rate in patients with luminal B subtype tumors (HR(+)/HER2(−)), slow growth rate in patients with luminal B subtype tumors (HR(+)/HER2(+)), and very slow growth rate in patients with luminal A subtype tumors (HR(+)/HER2(−))), the patient could be considered to be almost healthy, and she could be classified into the survival group. The consolidated mathematical growth model of the PT and sdMTSs of BC (CoMPaS) can calculate the total period of MTS(1-X) diagnosis and determine the time when patients may be considered healthy (Figure 1, Figure 2, Figure 3, Figure 4, Figure 5, Figure 6, Figure 7 and Figure 8; Table 1).

As a consequence, oncologists can determine the causes of BC appearance considering the knowledge of when the first tumor cell appeared, which can lead to the development of prevention methods. The relationship between the PT and sdMTSs can provide a deeper understanding of the BC process [2,3,4,5,6,7,8,9,10,11,12,13,14,15,20,21,36,37,38,39,40,41,73,74,75,76,77,78,79,80,81,82,83,84,85,86,87,88,89]. The calculation of the earliest diagnostic period can help with the assignment of early treatment and increase survival [82,83,84,85,86,87,88,89,100]. Clinics can plan procedures with optimal usage of resources and an understanding of when a patient will come to the hospital.

## 4. Materials and Methods

### 4.1. Consolidated Mathematical Growth Model of Primary Tumor and Secondary Distant Metastases (CoMPaS)

To describe the growth processes of PTs and sdMTSs at stages I and II, the CoMPaS model was developed. A detailed description and the limitations of CoMPaS, as well as the influence of the appearance of the first sdMTS on the survival prognosis of a patient, were provided previously. Figure 1 demonstrates the model in terms of the whole natural history of PT and sdMTS growth [21].

The whole natural growth history of the PT and sdMTSs includes the non-visible growth period of the PT, the visible growth period of the PT, the non-visible growth period of the sdMTSs, and the visible growth period of the sdMTSs [21]. The non-visible period of PT growth is from the appearance of the first tumor cell (diameter = 10 µm) until it reaches a visible size (diameter = 1–5 mm) [21]. The visible period of PT growth is from the time that it reaches a visible size (diameter = 1–5 mm) up to the time that it reaches pre-surgery size. The non-visible period of MTS growth can be calculated as the period from diagnosis (date of PT resection) to the time that at least one MTS reaches a visible size (diameter = 1–5 mm) [21]. The visible period of MTS growth can be calculated as the period from diagnosis of the visible size (diameter = 1–5 mm) to when it reaches lethal size (death) [21]. Thus, descriptions of the whole natural history of BC require building a consolidated mathematical BC growth model of the PT and secondary distant MTSs [21].

The following updated formulas illustrate the mathematical side of the CoMPaS [21]:

(1) $$\frac{dV}{dt}=\frac{\log2}{DT}V,t\leq DT\;\;\log2\left(\theta \frac{DT}{\log2}\right)V_{0},$$

(2) $$\frac{dV}{dt}=\theta \log V,t>DT\;\;\log_{2}\left(\theta \frac{DT}{\log2}V_{0}\right),$$

$$V\left(t=0\right)=V_{0},$$

(3) $$60=\log2^{N_{pt}}+\log2^{N_{mtsII}}+\log2^{N_{mtsII-vis}},$$

(4) $$TVDT_{non}=TVDT_{vis}=\frac{\log2_{days}^{N_{mtsII}}+\log2_{days}^{N_{mtsII-vis}}}{\log2^{N_{mtsII}}+\log2^{N_{mtsII-vis}}},$$

where $\frac{\log2}{DT}$ is the fraction of proliferative cell times, θ drives the linear phase (θ=1), *Npt* is the number of PT doublings, *NmtsII* is the number of doublings for the *non-visible* growth period of sdMTS, *Nmts-vis* is the number of doublings for the *visible* growth period of sdMTS, *TVDT* is the tumor volume doubling time, and 60 doublings represent the *whole nature growth history* of the PT and sdMTSs.

CoMPaS is based on an exponential growth model that consists of nonlinear and linear deterministic equations [21]. Available studies based on clinical data demonstrate exponential PT growth in patients with BC [36,37,38,39,44,45,46,47]. The growth rate of the PT in patients with BC is calculated via TVDT_PT_ [36,37,38,39,44,45,46,47]. An ultrasound-based diagnosis allows a better detection of the changes in PT sizes in patients with BC to calculate TVDT_PT_ [48,49,50,51,52,53,54].

The appearance of the first metastatic cell of the first sdMTS coincides with the 20th doubling of the PT of BC, which allows defining the non-visible growth period of the sdMTS and the initial period of sdMTS manifestation [7,14,15,21,55,56]. The appearance of the last metastatic cell of sdMTS coincides with the date of BC PT resection [21].

Available studies based on clinical data demonstrate exponential sdMTS growth between 1 mm and 60 mm in patients with BC [20,21,55,56,57,58,59,60,61,62]. The growth rate of sdMTSs in patients with BC is calculated via TVDT_MTS_ [57,58,59,60,61,62]. An ultrasound diagnosis allows a better detection of the changes in sdMTS sizes (liver, subcutaneous MTSs) in patients with BC to calculate TVDT_PT_ [48,49,50,51,52,53,54]. A computed tomography (CT) scan and magnetic resonance imaging (MRI) scan also allow a better detection of the changes in sdMTS sizes (lungs, brain) in patients with BC to calculate TVDT_PT_ [48,49,50,51,52,53,54].

The growth rate of sdMTSs in patients with BC (TVDT_MTS_) may correspond to (equal) the growth rate of the PT (TVDT_PT_) or may be 2–2.2–times higher [2,3,4,5,6,7,55,56]. The growth rate of secondary distant MTSs may be a rapid growth rate, an intermediate growth rate, or a slow growth rate [2,3,4,6,7]. The growth rate of the PT and sdMTSs in patients with BC can determine the survival forecast of these patients [2,3,4,5,6,7,41,42,62,63,64,65,66,67,68].

The model describes the following: (a) PT growth from 1 mm up to 60 mm (exponential growth of the PT) without/with MTSs in the lymph nodes; (b) a TVDT_PT_ from 10 days to 310 days; (c) sdMTSs growth from 1 mm up to 60 mm (exponential growth of the sdMTSs); (d) a TVDT_MTS_ from 10 days to 310 days. TVDT_MTS_ must be bigger than 10 days, but TVDT_MTS_ must be less than or equal to TVDT_PT_.

### 4.2. A Mathematical Model to Predict the Earliest Diagnostic Periods of Secondary Distant Metastases in Patients with ER/PR/HER2/Ki-67 Subtypes of Breast Cancer

The growth period of sdMTSs includes the non-visible growth period of sdMTSs, the visible growth period of sdMTSs, diagnostics, treatment, and patient death [21]. The non-visible growth period of sdMTSs consists of two periods: (1) the non-visible growth period of the sdMTS from the appearance of the first metastatic cell of the sdMTS to the date of PT resection (non-visible sdMTS(1)-I), and (2) the non-visible growth period of the sdMTS from the diagnosis (the date of PT resection) to the time when the visible size of at least one sdMTS can be diagnosed (non-visible MTS(1)-II) (Figure 1). It should be noted that the non-visible growth period of sdMTS(1)-II is described as the MTS-free period in other papers.

It is difficult to predict which metastatic cell (from the PT: 1 mm, 2 mm or 3 mm) will be the initial point of sdMTS growth (Figure 1). Considering prior assumptions, it is relevant to set the initial point as the *non-visible MTS(1-X) (years)* and the progression as the *Period of MTS(1-X) diagnosis* for the critical periods of *the earliest diagnosis* of the sdMTS (Figure 1).

The *natural* growth rate of the sdMTS may be similar to the *natural* growth rate of the PT of BC (Figure 1).

PTs of breast cancer were divided into five different groups depending on the ER/PR/HER2/Ki-67 subtypes and growth rates [49,50,52,53,54]:

Group I with a very slow growth rate of the PT, with subtype I of BC—luminal A = HR(+)/HER2(−) for HR-positive (ER+/PR+, ER+/PR−, or ER−/PR+) tumors, Ki-67 < 14% (TVDT_PT_ = 231–270 days);

Group II with a slow growth rate of the PT, with subtype II of BC—luminal B = HR(+)/HER2(+) for HR-positive (ER+/PR+, ER+/PR−, or ER−/PR+) and Her2-positive tumors, Ki-67 ≥ 14% (TVDT_PT_ = 196–230 days);

Group III with an intermediate growth rate of the PT, with subtype III of BC—luminal B = HR(+)/HER2(−) for HR-positive (ER+/PR+ or ER+/PR− or ER−/PR+) tumors, Ki-67 ≥ 14% (TVDT_PT_ = 166–195 days);

Group IV with an intermediate growth rate of the PT, with subtype IV of BC—HR(−)/HER2(+) for HER2-positive tumors, Ki-67 ≥ 14% (TVDT_PT_ = 136–165 days);

Group V with a rapid growth rate of the PT with subtype V of BC—HR(−)/HER2(−) for triple-negative tumors, Ki-67 ≥ 14% (TVDT_PT_ = 10–135 days).

However, the growth rate of the secondary distant MTS may be higher than the *natural* growth rate of the BC PT [2,3,4,6,7]. For the first time, the growth rates of secondary distant MTSs were divided into five groups depending on the ER/PR/HER2/Ki-67 subtypes and growth rates [49,50,52,53,54]: group I with subtype I of BC (luminal A = HR(+)/HER2(−)) for sdMTSs with very slow growth rates (TVDT_MTS_ = 231–270 days), group II with subtype II of BC (luminal B = HR(+)/HER2(+)) for sdMTSs with slow growth rates (TVDT_MTS_ = 196–230 days), group III with subtype III of BC (luminal B = HR(+)/HER2(−)) for sdMTSs with intermediate growth rates (TVDT_MTS_ = 166–195 days), group IV with subtype IV of BC (HR(−)/HER2(+)—HER2-positive tumors) for sdMTSs with intermediate growth rates (TVDT_MTS_ = 136–165 days), and group V with subtype V (HR(−)/HER2(−)—triple-negative tumors) of BC for sdMTSs with rapid growth rates (TVDT_MTS_ = 10–135 days) [2,3,4,6,7,20,22,23,24,25,26,40,47,49,52,53,54,69,70,71,72].

### 4.3. Limitations

The model does not describe or explain an appearance of the secondary distant MTSs (M1) in patients with stage T4N0-3M0.

### 4.4. Implementation Software

The application was built using Swift and references CoMPaS, where the input data consist of the following fields that a user (doctor) must fill: the *first* diagnostic data (date of diagnostics, diameter of the PT in mm) and the *second* diagnostic data (date of diagnostics, diameter of the PT in mm, subtype). As a result, the output data provide the following: prognosis (the *category* of prognosis: favorable, mid-favorable, unfavorable; the *number of months* before the manifestation of the sdMTSs) [21].

### 4.5. Calculation Method

The obtained results were calculated on a personal computer (PC) using Python 3.8.

## 5. Conclusions

The implementation of CoMPaS and the corresponding software tool offers fascinating prospects for personalized diagnostics and early treatment by detecting the earliest diagnostic period of sdMTSs in BC patients (T1-3N0-3M0 and ER/PR/HER2/Ki-67 subtypes) with regard to the eighth edition AJCC prognostic staging system for breast cancer and the growth rate of the PT and sdMTSs in BC [22,23,24,25,26,69,70,71,72,101,102,103,104,105,106]. Such mathematics-based approaches could better identify high-risk patients in the future and help prevent unnecessary treatments. Therefore, this approach could integrate diagnostic oncology more closely with outcome-oriented clinical decisions by increasing the survival of BC patients with sdMTSs (T1-3N0-3M0 and ER/PR/HER2/Ki-67 subtypes). These kinds of gains in efficiency will become increasingly important, given the growing demand for less toxic treatments and the discovery of almost healthy patients.

## 6. Patents

The predictor of the whole natural history of breast cancer (COMBREC): Certificate of the state registration of the computer program No. 2018612104. Date of the state registration in the register of computer programs: 12.02.2018. Authors: Neznanov AA, Tyuryumina EYa.

## Figures and Tables

**Table 1:** Diagnostic periods for sdMTSs in patients with ER/PR/HER2/Ki-67 (Estrogen Receptor/Progesterone Receptor/Human Epidermal growth factor Receptor 2/Ki-67 marker) subtypes of BC for stage T1cN0-3M0. TVDT—tumor volume doubling time.

	T1c (mm) 10 < d ≤ 20	T1c (mm) 10 < d ≤ 20	T1c (mm) 10 < d ≤ 20	T1c (mm) 10 < d ≤ 20	T1c (mm) 10 < d ≤ 20
**d_PT_ at surgery (mm)**	15.1	15.1	15.1	15.1	15.1
**PT_log(V)_**	31.7	31.7	31.7	31.7	31.7
**TVDT_PT_ (days)**	**80–110–135**	**136–150–165**	**166–180–195**	**196–210–230**	**231–250–270**
**TVDT_PT_ (days) mean**	**110**	**150**	**180**	**210**	**250**
	**V subtype**	**IV subtype**	**III subtype**	**II subtype**	**I subtype**
**Good prognosis**					**I subtype**
**Very slow growth rate**					**HR(+)/Her2(−)**
**TVDT_MTS_ (days)**				**230(+)**	**231–270**
**Mean TVDT_MTS_ (days)**				**230(+)**	**116–135**
**d_MTS_ (mm)**				9.0	9.0
**Non-visible MTS(1-X)-I**				7.41–14.80	7.43–8.69
**Non-visible MTS(1-X)-II**				11.19–18.58	11.23–13.13
**Visible MTS(1-X) (years)**				6.66–14.05	6.68–7.81
**Survival MTS(1-X) (years)**				17.84–25.23	17.91–20.93
**Period of MTS(1-X) diagnosis**				7.40	1.95
**Screening time**				12 mo	12 mo
**Intermediate prognosis**				**II subtype**	
**Slow growth rate**				**HR(+)/Her2(+)**	
**TVDT_MTS_ (days)**			**195(+)**	**196–230**	**196–230**
**Mean TVDT_MTS_ (days)**			**195(+)**	**98–115**	**98–115**
**d_MTS_ (mm)**			9.0	9.0	9.0
**Non-visible MTS(1-X)-I**			6.28–12.55	6.31–7.40	6.31–7.40
**Non-visible MTS(1-X)-II**			9.49–15.76	9.53–11.18	9.53–11.18
**Visible MTS(1-X) (years)**			5.65–11.91	5.67–6.65	5.67–6.65
**Survival MTS(1-X) (years)**			15.13–21.39	15.20–17.83	15.20–17.83
**Period of MTS(1-X) diagnosis**			6.27	1.70	1.70
**Screening time**			8 mo	8 mo	8 mo
**Intermediate prognosis**			**III subtype**		
**Intermediate growth rate**			**HR(+)/HER2(−)**		
**TVDT_MTS_ (days)**		**165(+)**	**166–195**	**166–195**	**166–195**
**Mean TVDT_MTS_ (days)**		**165(+)**	**83–97**	**83–97**	**83–97**
**d_MTS_ (mm)**		9.0	9.0	9.0	9.0
**Non-visible MTS(1-X)-I**		5.32–10.62	5.34–6.27	5.34–6.27	5.34–6.27
**Non-visible MTS(1-X)-II**		8.03–13.33	8.07–9.48	8.07–9.48	8.07–9.48
**Visible MTS(1-X) (years)**		4.78–10.08	4.80–5.64	4.80–5.64	4.80–5.64
**Survival MTS(1-X) (years)**		12.80–18.10	12.87–15.12	12.87–15.12	12.87–15.12
**Period of MTS(1-X) diagnosis**		5.31	1.46	1.46	1.46
**Screening time**		6 mo	6 mo	6 mo	6 mo
**Intermediate prognosis**		**IV subtype**			
**Intermediate growth rate**		**HR(−)/HER2(+)**			
**TVDT_MTS_ (days)**	**135(+)**	**136–165**	**136–165**	**136–165**	**136–165**
**Mean TVDT_MTS_ (days)**	**135(+)**	**68–82**	**68–82**	**68–82**	**68–82**
**d_MTS_ (mm)**	9.0	9.0	9.0	9.0	9.0
**Non-visible MTS(1-X)-I**	4.35–8.69	4.38–5.31	4.38–5.31	4.38–5.31	4.38–5.31
**Non-visible MTS(1-X)-II**	6.57–10.91	6.61–8.02	6.61–8.02	6.61–8.02	6.61–8.02
**Visible MTS(1-X) (years)**	3.91–8.25	3.93–4.77	3.93–4.77	3.93–4.77	3.93–4.77
**Survival MTS(1-X) (years)**	10.48–14.81	10.54–12.79	10.54–12.79	10.54–12.79	10.54–12.79
**Period of MTS(1-X) diagnosis**	4.34	1.46	1.46	1.46	1.46
**Screening time**	5 mo	5 mo	5 mo	5 mo	5 mo
**Poor prognosis**	**V subtype**				
**Rapid growth rate**	**HR(−)/HER2(−)**				
**TVDT_MTS_ (days)**	**10–135**	**10–135**	**10–135**	**10–135**	**10–135**
**Mean TVDT_MTS_ (days)**	**40–67**	**40–67**	**40–67**	**40–67**	**40–67**
**d_MTS_ (mm)**	9.0	9.0	9.0	9.0	9.0
**Non-visible MTS(1-X)-I**	0.55–4.34	0.55–4.34	0.55–4.34	0.55–4.34	0.55–4.34
**Non-visible MTS(1-X)-II**	0.26–6.56	0.26–6.56	0.26–6.56	0.26–6.56	0.26–6.56
**Visible MTS(1-X) (years)**	0.29–3.90	0.29–3.90	0.29–3.90	0.29–3.90	0.29–3.90
**Survival MTS(1-X) (years)**	0.55–10.47	0.55–10.47	0.55–10.47	0.55–10.47	0.55–10.47
**Period of MTS(1-X) diagnosis**	6.30	6.30	6.30	6.30	6.30
**Screening time**	3 mo	3 mo	3 mo	3 mo	3 mo
***Total period MTS(1-X) diagnosis***	10.64	13.07	15.49	18.32	12.87

*d_PT_* at surgery (mm)—the mean size (mm) of the PT at surgery (resection of the PT) for each stage (T1, T2, T3), obtained from Table 1 of [15]; *PT_log(V)_*—the number of doublings of the PT at surgery (resection of the PT); *TVDT_PT_*—the mean tumor volume doubling time of the PT (days) at surgery (resection of the PT) [20,21,44,45,46,47,48,49,50,51,52,53,54,55,56]; *TVDT_MTS_*—the mean tumor volume doubling time of the sdMTS (days) [20,21,57,58,59,60,61,62,63,64,65,66,67,68]; *d_MTS_*—the mean size (mm) of the sdMTS at the diagnosis period; *Non-visible MTS(1-X)-I (years), Non-visible MTS(1-X)-II (years), Visible MTS(1-X) (years) and Period of MTS(1-X) diagnosis*—see Figure 1. *Survival MTS(1-X) (years)*—the survival (lifetime) can be calculated as the period between the date of diagnosis (using the tumor-node-metastasis (TNM) staging system) and the date of patient death. The survival of BC patients includes both the non-visible and visible growth periods of the sdMTS (the first, intermediate, or last sdMTS from the PT); *Total period of MTS(1-X) diagnosis (years)*—sum of the periods of the MTS(1-X) diagnosis (periods of rapid growth rate, intermediate growth rate, and slow and very slow growth rates). *I subtype*—Luminal A, HR(+)/Her2(−), Ki-67 < 14% for HR-positive (ER+/PR+ or ER+/PR− or ER−/PR+) tumors [22,23,24,25,26,69,70,71,72]; *II subtype*—Luminal B, HR(+)/Her2(+), Ki-67 ≥ 14% for HR-positive (ER+/PR+ or ER+/PR− or ER−/PR+) and Her2-positive tumors [22,23,24,25,26,69,70,71,72]; *III subtype*—Luminal B, HR(+)/Her2(−), Ki-67 ≥ 14% for HR-positive (ER+/PR+ or ER+/PR− or ER−/PR+) tumors [22,23,24,25,26,69,70,71,72]; *IV subtype*—HER2-positive, HR(−)/Her2(+), Ki-67 ≥ 14% [22,23,24,25,26,69,70,71,72]; *V subtype*—Triple Negative, HR(−)/Her2(−), Ki-67 ≥ 14% [22,23,24,25,26,69,70,71,72].
